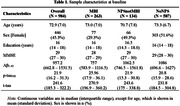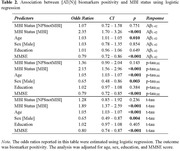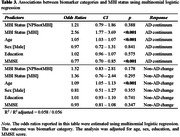# Screening for preclinical and prodromal Alzheimer disease clinical trials: predicting NIA‐AA research framework ATN category by leveraging neuropsychiatric symptoms

**DOI:** 10.1002/alz.092467

**Published:** 2025-01-09

**Authors:** Zahinoor Ismail, Rebeca Leon, Dylan X. Guan, Eric E. Smith

**Affiliations:** ^1^ Hotchkiss Brain Institute, University of Calgary, Calgary, AB Canada; ^2^ University of Calgary, Calgary, AB Canada; ^3^ Department of Clinical Neurosciences and Hotchkiss Brain Institute, University of Calgary, Calgary, AB Canada

## Abstract

**Background:**

As disease‐modifying therapies for Alzheimer disease (AD) emerge, the need for earlier detection becomes increasingly important. Biomarker confirmation is expensive and invasive, only implemented for persons at risk. Risk is usually assigned based on cognitive decline. Accordingly, detection at NIA‐AA Research Framework Stage 2 (preclinical disease) or early Stage 3 (prodromal) is suboptimal. Whether incorporating neuropsychiatric symptoms (NPS) helps with detection remains in equipoise. However, by leveraging risk associated with later‐life emergent and persistent behavioral changes, Mild Behavioral Impairment (MBI) has demonstrated associations with lower amyloid‐β (Aβ) and higher tau levels in blood and CSF. Whether MBI can categorically predict prevalent AD is unclear. Here, we examined cross‐sectional associations between MBI and cerebrospinal fluid (CSF) [AT(N)] biomarker categories.

**Method:**

Dementia‐free participants (n=984) from the Alzheimer's Disease Neuroimaging Initiative were included. Using the validated two‐thirds visit method to operationalize symptom persistence, NPS (identified using the Neuropsychiatric Inventory or Neuropsychiatric Inventory Questionnaire) were categorized into three NPS groups: 1) NoNPS; 2) NPS not meeting MBI criteria (NPSnotMBI); and 3) MBI. Consistent with the [AT(N)] framework, three biomarker profiles were described: 1) normal AD biomarkers; 2) AD continuum (low CSF Aβ1‐42); and 3) non‐AD pathologic change (normal Aβ, elevated CSF p‐tau181 and/or t‐tau). Gaussian Mixture Modeling (GMM) was used to determine biomarker positivity thresholds with Winsorization of extreme values. Logistic regression modeled associations between MBI status and CSF biomarker positivity, adjusted for age, sex, education, and MMSE score. Multinomial logistic regression modeled associations between MBI status and [AT(N)] biomarker profile.

**Result:**

Table 1 shows demographics. Compared to the NoNPS group, MBI was significantly associated with Aβ positivity (OR=2.35, 95%CI=1.70–3.26, p<0.001), p‐tau181 positivity (OR=2.15, 95%CI=1.56–2.96, p<0.001), and t‐tau positivity (OR=1.89, 95%CI=1.37–2.59, p<0.001), while NPSnotMBI was not (Table 2). Multinomial logistic regression indicated a significant association between MBI+ status and the AD continuum category (OR=2.56, 95%CI=1.77–3.69, p<0.001), but not non‐AD pathologic change (Table 3).

**Conclusion:**

In dementia‐free older individuals, MBI predicts Aβ‐positivity and AD+ status over non‐AD pathological change; no associations were found with NPSnotMBI. Incorporating MBI into AD trial screening can improve disease detection and reduce screen failures.